# Direct Precipitation of Lignin Nanoparticles from Wheat Straw Organosolv Liquors Using a Static Mixer

**DOI:** 10.3390/molecules25061388

**Published:** 2020-03-18

**Authors:** Stefan Beisl, Johannes Adamcyk, Anton Friedl

**Affiliations:** Institute of Chemical, Environmental and Bioscience Engineering, TU Wien, 1060 Vienna, Austria; johannes.adamcyk@tuwien.ac.at (J.A.); anton.friedl@tuwien.ac.at (A.F.)

**Keywords:** lignin, nanoparticles, microparticles, biorefinery, wheat straw, direct precipitation, organosolv

## Abstract

Micro- and nanosize lignin shows improved properties compared to standard lignin available today and has been gaining interest in recent years. Lignin is the largest renewable resource with an aromatic skeleton on earth but it is used for relatively low-value applications. Lignin in micro- to nanoscale; however, could facilitate rather valuable applications. Current production methods consume high amounts of solvents for purification and precipitation. The process investigated in this work uses the direct precipitation of lignin nanoparticles from organosolv pretreatment extract in a static mixer and can reduce solvent consumption drastically. The pH value, ratio of antisolvent to organosolv extract and flowrate in the mixer were investigated as precipitation parameters in terms of the resulting particle properties. Particles with dimensions ranging from 97.3 to 219.3 nm could be produced, and at certain precipitation parameters, carbohydrate impurities reach values as low as in purified lignin particles. Yields were found independent of the precipitation parameters with 48.2 ± 4.99%. Results presented in this work can be used to optimize precipitation parameters with emphasis on particle size, carbohydrate impurities or the solvent consumption.

## 1. Introduction

Many petrochemicals are produced from conventional crude oil-fed refineries, whereas it is anticipated that in the future, many products and chemicals will be produced from biorefineries fed with lignocellulosic biomass such as agricultural residuals [1]. This renders the term “waste”, in the context of biomass processing terminology, obsolete as each production stream has the potential to be converted into a by-product or energy rather than waste [2]. However, lignin, as the second-most abundant biopolymer on Earth after cellulose, is underutilized in first-generation cellulosic projects and most of this lignin is currently used as an energy source. However, economic analysis has proved that the use of biomass for energy applications alone, in many cases, is not economically viable and utilization of the entire biomass through multiple processes is needed to improve its economic benefits [3]. Only about 40% of the produced lignin is needed to cover a biorefinery’s internal energy demand [4,5]. Hence, a major part of the produced lignin is available to increase the revenue of a biorefinery in addition to the valorization of the carbohydrate fractions.

Lignin is a highly-irregular branched polyphenolic polyether, consisting of the primary monolignols, *p*-coumaryl alcohol, coniferyl alcohol and sinapyl alcohol, which are connected via aromatic and aliphatic ether bonds [6]. Roughly three different types of lignins can be distinguished: Softwood lignins are comprised almost solely of coniferyl alcohol, hardwood lignins of both coniferyl and sinapyl alcohol and grass lignins of all three types [7]. The high complexity and inhomogeneity of the lignin structure is, in many cases, even further increased by currently applied pretreatment technologies and adds additional challenges for lignin’s downstream processing and valorization [8,9]. Compared to other pretreatment technologies, the organosolv process used in this work extracts relatively pure, low-molecular weight lignin from biomass. This lignin shows a minimum of carbohydrate and mineral impurities and facilitates lignin applications with higher value than heat and power generation [10].

An approach to overcome this high complexity and inhomogeneity is the production and application of nanostructured lignin. Nanostructured materials, especially in the 1–100 nm range, offer unique properties due to their increased surface area [11], while their important chemical and physical interactions are governed by surface properties. Hence, a nanostructured material can have considerably different properties than a larger-dimensional material of the same composition [12]. Therefore, the preparation of lignin nanoparticles and other nanostructures has gained interest among researchers during the last few years.

Lignin nano- and microparticles have diverse potential applications, ranging from improving mechanical properties of polymer nanocomposites [11,12,13,14,15], bactericidal [12] and antioxidant properties [16,17] and impregnations [18] to drug carriers for hydrophobic and hydrophilic substances [19,20,21,22,23,24,25]. Additionally, a carbonization of lignin nanostructures can lead to high-value applications such as use in supercapacitors for energy storage [26]. Particles in their native form can also be used to stabilize oil-water emulsion for a multitude of applications [27,28,29]. Furthermore, first attempts for upscaling a precipitation process in tetrahydrofuran–water solvent systems are under investigation [30]. However, most production methods published to date have a very high solvent consumption in common. Vast amounts of solvents are needed to purify the lignin prior to precipitation, the precipitation itself and the downstream processing [31].

This work is focused on the direct precipitation of lignin nanoparticles from organosolv pretreatment extracts (OSE) in a wheat straw biorefinery potentially reducing the solvent consumption of the entire process. Therefore, it is also tackling the challenge that may be associated with the large-scale commercialization of lignin nano- and microparticles where the particle precipitation should be integrated into, or be in harmony with, lignin extraction processes so that lignin polymers and LNPs can be produced by industry on a large commercial scale [32].

The precipitation is conducted in a static mixer, which results in smaller particles compared to a batch precipitation [33]. It combines the most commonly used precipitation methods, solvent shifting and pH shifting, and reduces lignin solubility by decreasing the solvent concentration and lowering the pH value [31]. The degree of lignin supersaturation [34], hydrodynamic conditions prevailing during the process [35] and the pH value of the particles’ surrounding fluid [36] are important parameters influencing the final particle size and behavior. These mentioned process conditions are investigated by varying the precipitation parameters pH value, ratio of antisolvent to OSE and the flowrate in the static mixer. The resulting particles were investigated in terms of particle size, stability, carbohydrate impurities and the yield of the process. The best precipitation parameters were identified and a comparison with the precipitation of previously purified and re-dissolved lignin was made.

## 2. Results and Discussion

### 2.1. Ratio of Antisolvent/Organosolv Extract

**Figure 1:**
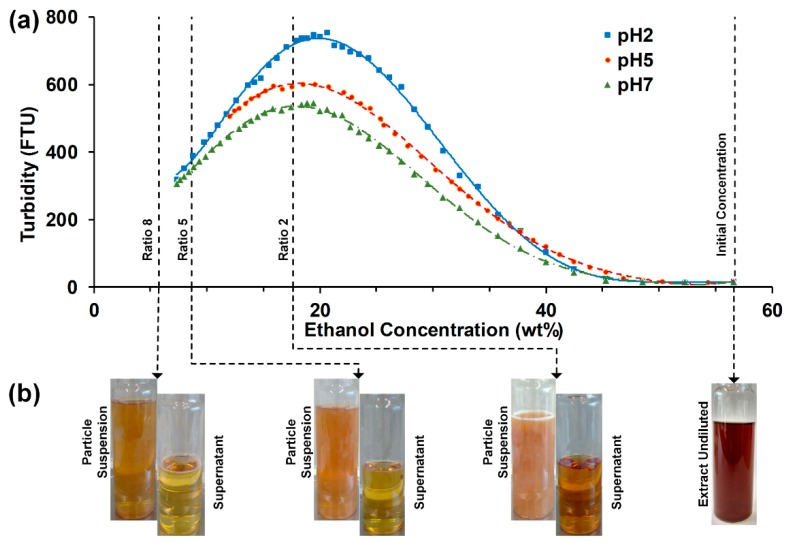
(**a**) Turbidity against ethanol concentration in solution/suspension. The ethanol concentration was gradually lowered by adding antisolvent at different pH values to the organosolv extract in a stirred vessel. (**b**) Pictures of particle suspensions and supernatants after centrifugation originate from precipitations in the static mixer with pH 5 antisolvent and a flowrate of 112.5 mL/min.

Lignin solubility is strongly dependent on the ethanol concentration in ethanol-water solvent mixtures and type of lignin [37]. To determine the necessary final ethanol concentration in the precipitation process and therefore the ratio of antisolvent to OSE, the turbidity was measured in dependency of the ethanol concentration (see Figure 1). Pure water and water/sulfuric acid mixtures were gradually added to OSE in a stirred flask with an initial ethanol concentration of 56.7wt%. In order to stay within the measuring range of the turbidity meter, the initial OSE was diluted by a factor of 1:6 by mass while maintaining the initial ethanol concentration. The neat lignin concentration of 7.35 g/kg was; therefore, reduced to 1.23 g/kg. This might lead to a slight shift of the turbidity maxima towards lower ethanol concentrations, since the solubility limit is reached at lower ethanol concentrations. The points of interest are the maxima of the turbidity curves to determine the minimum antisolvent/OSE ratios required for precipitation. The turbidity maxima are reached at 19.9, 18.1 and 17.9wt% for adding antisolvent with a pH of 2, 5 and 7, respectively. The lowest antisolvent/OSE ratio for the precipitation experiments was therefore set to 2, resulting in a final ethanol concentration in the suspension of 17.6wt%. Further investigated ratios were set to 5 and 8, resulting in a final ethanol concentration of 8.7 and 5.7wt%, respectively, to increase the supersaturation of the lignin. The shift in the maxima of the turbidity towards higher ethanol concentrations for decreasing pH values indicate a decreasing lignin solubility with decreasing pH values. However, the lowest antisolvent pH value applied for the precipitation experiments in the static mixer was set to 3 instead of 2, due to an isoelectric point at a pH value of around 2.5 that was identified in the ζ-potential measurements.

### 2.2. Particle Size

**Figure 2:**
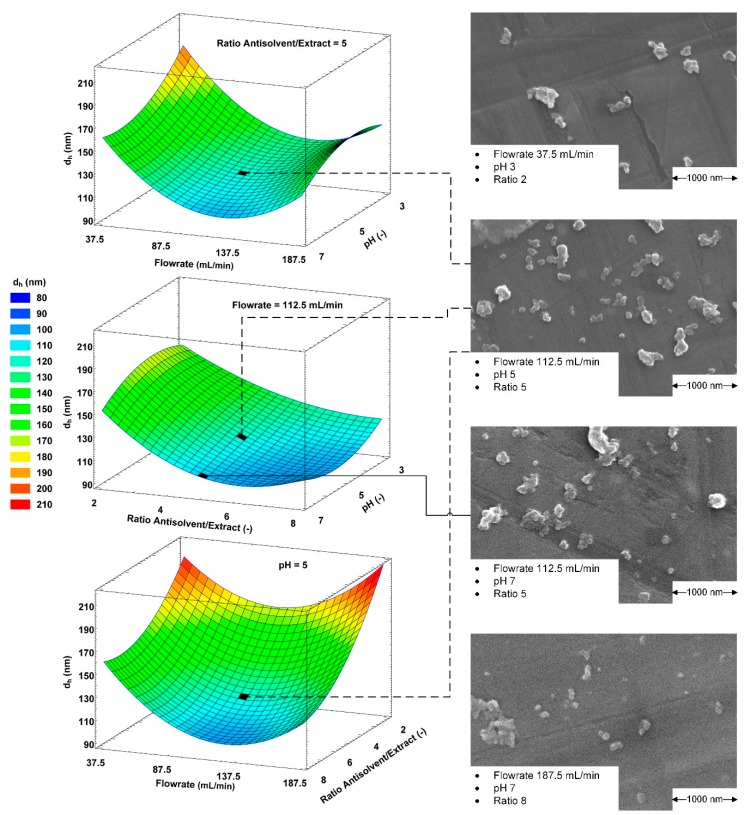
Effect of independent variables’ interaction on the hydrodynamic diameter of the resulting particles and SEM images of selected precipitation parameters.

The independent variables antisolvent pH value, flowrate in the static mixer and antisolvent/OSE ratio were investigated in terms of the resulting particle hydrodynamic diameter (HD). The resulting particle suspensions were measured via dynamic light scattering (DLS) directly after precipitation in two conditions: undiluted and in a 1:100 dilution with water. After correcting the viscosity and refractive index for the undiluted samples, the HDs for both dilution conditions were compared with a paired t-test and showed significantly equal results for both conditions. The results shown in Figure 2 are based on the HDs obtained by diluted measurements.

The resulting HDs span from 97.3 to 219.3 nm. The smallest HD is reached in precipitations with an antisolvent/OSE ratio of 6.29, pH 7 and a flowrate of 132.06 mL/min. The particles with the highest HD result from an antisolvent/OSE ratio of 2, pH 4.93 and flowrate of 187.5 mL/min.

The particles’ HD shows a strong dependency on the flowrate with minima between 107.25 and 138.0 mL/min depending on pH and ratio. This behavior might originate from changing flow conditions influencing the balance of primary nucleation and agglomeration by changing the supersaturation of lignin and the collision rate of the resulting particles. At low flowrates, the supersaturation is comparably low and larger particles are formed. With increasing flowrates, the mechanical mixing energy input and supersaturation of the lignin increases, leading to smaller particles. However, further increased supersaturation leads to higher collision and agglomeration rates [34]. A correlation between residence time in the static mixer and the particle size was not evident.

A similar behavior can be observed for the antisolvent/OSE ratio. HDs decrease with increasing ratios due to higher supersaturation coherently increasing nucleation rates. For example, at a constant pH value of 5 and flowrate of 112.5 mL/min, the particles’ HD decreases from 172.9 to 117.3 and 101.7 nm for ratios of 2, 5 and 8, respectively. The mechanical energy input; however, does not increase due to the constant flowrate. Therefore, particle collision rates are only dependent on the particle concentrations. Hence, higher antisolvent/OSE ratios coherently lead to lower agglomeration [36].

The pH value shows the lowest influence of the investigated variables on the HD. The HD increases from 104.0 to 131.2 nm by decreasing the antisolvent pH value from 7 to 3 at a constant antisolvent/OSE ratio of 5 and a flowrate of 112.5 mL/min. The increased HD at low pH values might be explained by the ζ-potential of the particles, which decreases towards pH values of 3 and reaches the isoelectric point at pH values of around 2.5.

**Figure 3:**
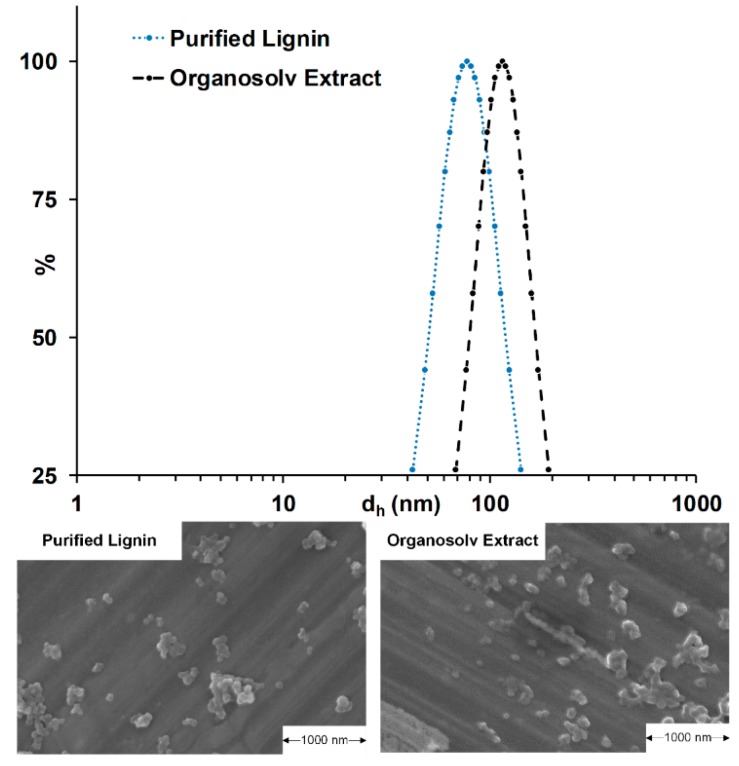
Hydrodynamic diameter distributions and SEM images of lignin particles precipitated directly from organosolv extract and from purified lignin (PL) solutions. Precipitation parameters used were pH 7, antisolvent/exctract ratio of 5 and a flowrate of 112.5 mL/min in the static mixer.

The OSE contains not only lignin but also components, such as carbohydrates, acetic acid and different degradation products, which must be considered as impurities during the precipitation process. To investigate the influence of these impurities, lignin from applied OSE was purified and dissolved in an aqueous ethanol solution with an ethanol concentration of 56.7wt%, equal to the neat OSE. The purified lignin (PL) solubility reached its limit at a concentration of 6.65 g/kg, which is lower than the lignin concentration of 7.35 g/kg in the OSE. Therefore, the OSE was diluted to the equal lignin concentration at constant ethanol concentration. The precipitation parameters were set to pH 7, ratio 5 and a flowrate of 112.5 mL/min, which represents the closest experimental point to the calculated parameters for the smallest particles. The HD distributions and SEM images of the precipitation directly from OSE and of the dissolved PL are shown in Figure 3. The PL precipitation results in an HD of 77.62 ± 2.74 nm, whereas the precipitation directly from the OSE leads to a higher HD of 102.7 ± 7.75 nm. A comparable result was achieved by Richter et al. [38] with organosolv lignin dissolved in acetone and precipitation leading to particles with approximately 80 nm in diameter.

The SEM images show only minor differences and show both separated particles. The SEM images were taken of the precipitate after the ultra-centrifugation at 288,000× *g* and influences of such high centrifugal forces on the particle shape and size cannot be excluded. However, based on the DLS results, the direct precipitation is resulting in higher HDs compared to the PL. Exact reasons need to be further investigated but include the influence of the impurities in the OSE and possible fractionation of the PL regarding their molecular mass in its first precipitation and purification step [39].

### 2.3. Yield

The precipitation yields were found to be independent of the precipitation parameters and had an average value of 48.2 ± 4.99%. The standard deviation was rather high, but the values were normally distributed. For comparison, Tian et al. [40] could reach yields between 41.0% and 90.9% using a dialysis method applying dimethyl sulfoxide as solvent for poplar, lodgepole pine and corn stover lignin and water as antisolvent. Furthermore, this work represents the most comparable process found in literature because a whole process chain from the raw material to the final lignin particles including impurities was shown. Yearla et al. [41] showed a process with yields from 33% to 63% by adding lignin/acetone/water mixtures rapidly in water.

The environmental impacts of this rather low precipitation on a whole biorefinery and possible improvements were addressed by another work of our group [42].

### 2.4. Carbohydrate Impurities

In addition to lignin, the OSE also contains carbohydrates as a major source of impurities during the precipitation. In terms of concentration, the total carbohydrate content in the extract amounts to 10.2% of the lignin content. These carbohydrates might lead to undesired growth of microorganisms in final applications and a reduction of such is therefore necessary. Therefore, the resulting precipitate after centrifugation and freeze drying was analyzed in terms of its carbohydrate content.

**Figure 4:**
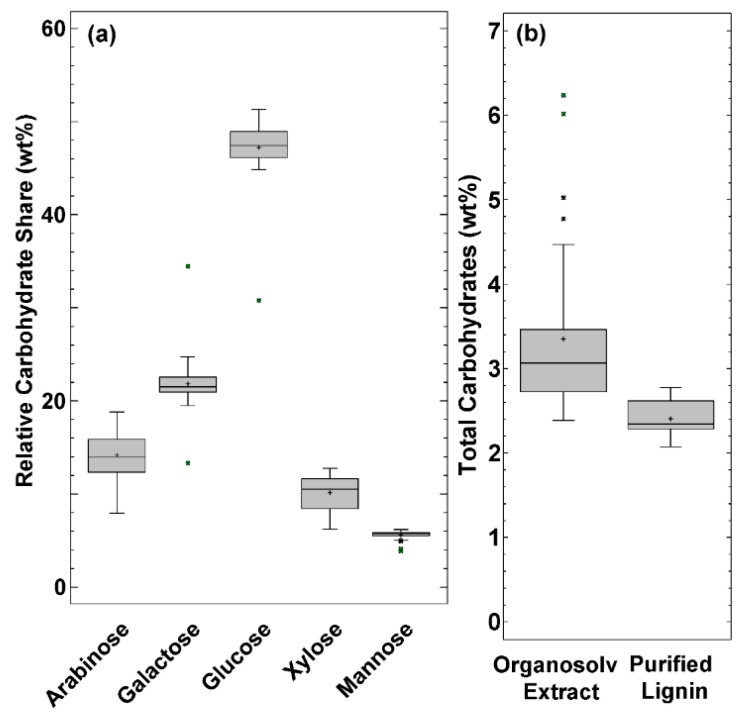
(**a**) Box plot of the relative carbohydrate share found in the 34 single experiments. (**b**) Box plot of the total carbohydrate content in the direct precipitate from organosolv extracts and in the purified lignin (PL). The + indicates the arithmetic mean.

The relative share of the carbohydrates is shown in Figure 4a. Glucose, with a relative share of 47.2 ± 3.36%, is the predominant carbohydrate compound in the precipitate. Figure 4b compares the carbohydrate concentrations found in the precipitate of the direct OSE experiments to the PL precipitates. The total carbohydrate content in the PL is 2.41 ± 0.25wt% and seems to be covalently bound to the lignin. The lowest carbohydrate content found within all direct OSE precipitates was 2.39wt%, which is in the concentration range of the PL. This indicates that certain precipitation parameters allow a precipitation of almost pure lignin in terms of carbohydrates dissolved in the OSE remaining on the particles.

**Figure 5:**
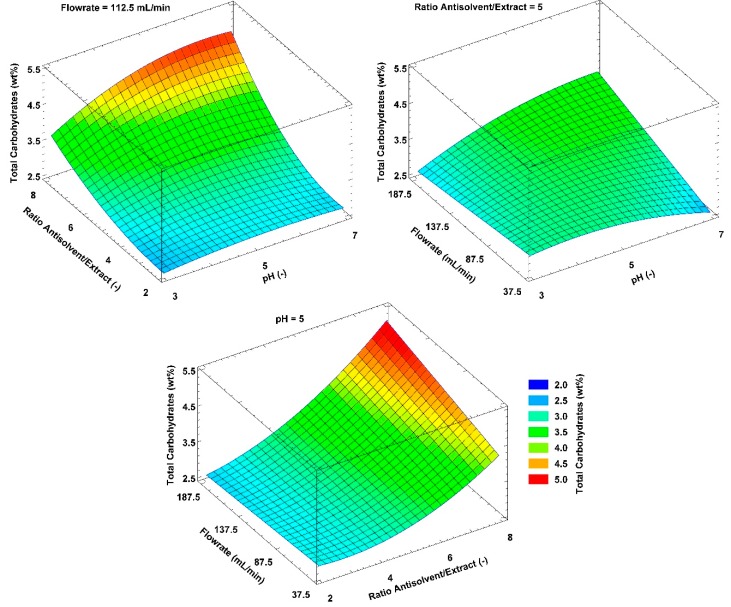
Effect of independent variables interaction on total carbohydrate content of the resulting dry precipitate.

To further investigate the behavior of carbohydrate impurities, their concentrations were correlated to the independent variables used in the precipitation analysis. However, a correlation between particle size and calculated surface area was not evident. Figure 5 shows the results of the carbohydrate concentration correlations. A maximum of 6.09wt% is gained at antisolvent/OSE ratio of 8, pH 7 and flowrate 187.5 mL/min and a minimum at 2.35wt% gained at antisolvent/OSE ratio of 2, pH 3 and flowrate 187.5 mL/min. These values are in a comparable range with the results of Huijgen et al. [43], who achieved carbohydrate contents in precipitated wheat straw organosolv lignins of 0.4 to 4.9wt% with treatment temperatures between 190 to 210 °C. The higher temperatures compared to 180 °C used in this work; however, favor the carbohydrate cleavage and lead to lower carbohydrate impurities.

In contrast to the implication that a higher dilution factor would decrease the carbohydrate content, the carbohydrate concentration increases with increasing antisolvent-to-extract ratio. The carbohydrate concentrations for a ratio of 2 are between 2.35 and 2.80wt% for precipitations with pH 3, a flowrate of 187.5 mL/min and pH 4.79 and a flowrate of 37.5 mL/min, respectively. For a ratio of 8, a concentration minimum of 3.47wt% and a maximum of 6.10wt% can both be found at a flowrate of 187.5 mL/min and a pH value of the antisolvent of 3 and 7, respectively.

A contradictory behavior is noticeable with increasing flowrate, which either contributes to a decreasing or increasing carbohydrate content in the precipitate depending on the pH and antisolvent/OSE ratio parameters. For a combination of pH 3 antisolvent and a ratio of 2, the carbohydrate concentration decreases from 2.72 to 2.35wt% by increasing the flowrate from 37.5 to 187.5 mL/min. On the other hand, by increasing the flowrate by 150.0 mL/min for the pH value und ratio combination of 5 and 8, respectively, the carbohydrate content increases from 4.18 to 5.21wt%.

The pH value shows a rising influence at increasing antisolvent/OSE ratios and flowrates. The carbohydrate concentration at otherwise constant precipitation parameters can be reduced by up to 43% by changing the antisolvent pH value. This maximum reduction is gained at an antisolvent/OSE ratio of 8 and a flowrate of 187.5 mL/min and the carbohydrate content can be reduced from 6.09 to 3.47wt% by changing the pH value from 7 to 3.

### 2.5. Stability

**Figure 6:**
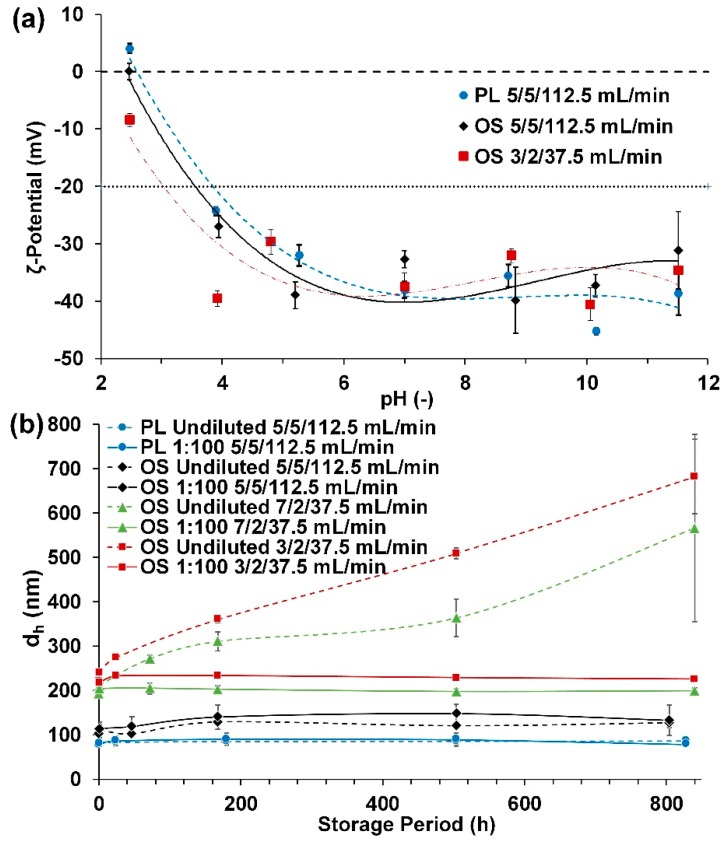
(**a**) ζ-potential over a pH range of 2.5 to 11.5; (**b**) long-term particle stability, where storage and precipitation parameters are labeled in following order: Type of particles (purified lignin (PL) and organosolv extract (OS)), storage condition (undiluted and 1:100 dilution), pH value, antisolvent/OSE ratio and flowrate.

Long-term particle stability was investigated, including their ζ-potential, as this is an important indicator for the stability. The ζ-potential was measured in a pH range of 2.5 to 11.5 and is shown in Figure 6a. The particles show a high stability between pH 4 and 11 with ζ-potentials of around −30 to −45 mV. When pH values reach below 4, the ζ-potential starts to increase and attains the isoelectric point at around 2.5. At elevated pH values above 11, lignin particle dissolution was observed. However, no major differences between different precipitation parameters as well as between the PL particles and the direct precipitated particles were evident.

Furthermore, particle behavior and stability are strongly dependent on their surrounding fluid. However, ζ-potential measurements are not possible in environments containing high amounts of impurities like those present in the neat suspensions, while those impurities can have an influence on the particles’ long-term stability. Therefore, stability was determined by long time storage experiments. Particle suspensions were stored in neat suspension as well as in a 1:100 dilution with pure water in order to investigate the influence of the impurity concentrations. The HD of the neat suspension was determined in a 1:100 dilution, which was made immediately before measurement. Figure 6b shows the particle size within a five-week storage period. The PL particles and the directly-precipitated lignin particles at the precipitation parameters pH 5, ratio 5 and a flowrate of 112.5 mL/min showed a stable behavior within five weeks in both storage conditions. PL particles increased their HD after five weeks to only 112% and 101% based on their initial particle size for storage in neat suspension and in a 1:100 dilution, respectively. Directly precipitated particles at equal precipitation parameters showed an increase to 124% and 130% for storage in a neat suspension and in 1:100 dilution, respectively. For particles precipitated at ratios of 2, the stability was strongly dependent on the storage conditions. For the precipitation parameters ratio 2, flowrate 37.5 mL/min and pH 3 and 7, the 1:100 diluted samples showed a stable HD within five weeks, whereas particles in the neat suspensions more than doubled their HD.

With consideration to the unchanging ζ-potential at different precipitation parameters and the improved long-term stability of particles precipitated with low ratios and, therefore, high impurity concentrations in the surrounding fluid, the long-term stability might be increased by exchanging or separating the impurities from the precipitated particles. Furthermore, a previous study showed that particles can be dried, stored and dispersed in water when needed, providing a further potential for long-term storage [33].

## 3. Materials and Methods

### 3.1. Materials

The wheat straw used was harvested in 2015 in the state of Lower Austria and stored under dry conditions until use. The particle size was reduced in a cutting mill, equipped with a 5 mm mesh, before pretreatment. The composition of the dry straw was 16.1wt% lignin and 63.1wt% carbohydrates consisting of arabinose, glucose, mannose, xylose and galactose. Ultra-pure water (18 MΩ/cm) and ethanol (Merck, Darmstadt, Germany, 96vol%, undenatured) was used in the organosolv treatment, and sulfuric acid (Merck, Darmstadt, Germany, 98%) was additionally used in the precipitation steps.

### 3.2. Organosolv Pretreatment

The organosolv pretreatment was conducted as previously described in Beisl et al. [33]. In brief, wheat straw was treated at a maximum temperature of 180 °C for 1 h in 60wt% aqueous ethanol. Residual particles were separated by centrifugation. The extract composition can be found in Table S1.

### 3.3. Precipitation

The precipitation setup used is described in Beisl et al. [33]. The batch setup used in Section 2.1 consists of a temperature-controlled 250 mL beaker with a magnetic stirrer and a syringe pump for addition of the antisolvent via an antidiffusion tip. The setup used in all other experiments consists of two syringe pumps, a static mixer (Striko, Wiehl, Germany) and a stirred collection vessel. The stirrer speed in the collection vessel was set to 375 rpm. The acidified antisolvents with pH values of 3 and 5 were adjusted with sulfuric acid and the pH 7 antisolvent was pure water. The particles were separated from the suspension after precipitation in a ThermoWX-80 + ultracentrifuge (Thermo Scientific, Waltham, MA, USA) at 288,000× *g* for 60 min. The supernatant was decanted, and the precipitate was freeze-dried. For the purified lignin (PL), lignin from the same extraction process was precipitated and purified by repeated sonication 10 times, centrifugation and replacement of the supernatant with water. The PL was freeze-dried and then dissolved in an ethanol/water mixture at equal ethanol concentrations compared to neat OSE. This artificial extract was used for comparison to the direct precipitation.

### 3.4. Design of Experiments

The experiments’ design and statistical evaluation of their results were conducted using the software Statgraphics Centurion XVII (Statpoint Technologies, Inc., Warrenton, VA, USA). A face-centered central composite design, including three center points with one full repetition (34 single experiments), was applied for the precipitation parameters flowrate in the static mixer, antisolvent pH value and the volume ratio of antisolvent to OSE. Flowrate levels in the static mixer were set to 37.5, 112.5 and 187.5 mL/min. The antisolvent to extract volume ratios were set to 2, 5 and 8, while the antisolvent pH values were 3, 5 and 7. The significance level was set to α = 0.05 in all statistical tests.

The results from the face-centered central composite design were used to describe the effects of the independent variables with a cubic model approach. High coefficients of determination were achieved for the carbohydrate content (R^2^ 0.89/Adj. R^2^ 0.87) and the particle size (0.92/0.88). The results from the analysis of variance are given in Table S2. However, the model approach was not applicable to the responses’ yield and stability and; therefore, they were evaluated conventionally as shown in Section 2.3 and Section 2.5. The used factors and resulting coefficients can be seen in Table S3. Non-significant factors were removed from the model stepwise and were not considered in the results.

### 3.5. Characterization

The ethanol concentration-dependent turbidity of the particle suspension was determined with a Hach 2100Qis (Hach, Loveland, CO, USA). To stay within the calibration range, the extract was diluted 1:6 by volume with ethanol/water to maintain the neat ethanol concentration of the extract. Water or sulfuric acid/water mixtures were gradually added to a stirred vessel filled with the diluted extract and measured after each addition.

The hydrodynamic diameter (HD) of the particles was measured using dynamic light scattering (DLS) (ZetaPALS, Brookhaven Instruments, Holtsville, NY, USA). The measurements were carried out in the particle suspension directly after precipitation—both undiluted and in a 1:100 dilution with pure water. Undiluted measurements were corrected by their viscosity and refractive index of the gained supernatant after centrifugation. For long-term stability tests, the particles were stored at 8 °C, but measured at 25 °C.

The ζ-potential was investigated with a ZetaPALS (Brookhaven Instruments, Holtsville, NY, USA). Dried particles were dispersed in water at an approximate concentration of 20 mg/L and aged 24 h before the measurement. Each measurement was composed of five runs, with 30 sub-runs each, and was conducted at 25 °C.

Freeze-dried particles were dispersed in hexane, spread on a sample holder and investigated in a scanning electron microscope (SEM) (Quanta 200 FEGSEM, Fei, Hillsboro, OR, USA). The samples were sputter coated with 4 nm Au/Pd (60/40wt%) before analysis.

The carbohydrate content was determined by the sample preparation following the National Renewable Energy Laboratory (NREL) laboratory analytical procedure (LAP), “Determination of Structural Carbohydrates and Lignin in Biomass” [44], but samples were not neutralized after hydrolysis was conducted. A Thermo Scientific ICS-5000 HPAEC-PAD system (Thermo Scientific, Waltham, MA, USA) with deionized water as the eluent was used for determining arabinose, glucose, mannose, xylose and galactose.

The yield was determined by the difference in dry matter content of the particle suspension directly after precipitation and of the supernatant of the particle suspension after centrifugation.

## 4. Conclusions

The influence of the precipitation parameters pH value, ratio of antisolvent to organosolv extract and flowrate in the mixer were investigated in terms of the resulting particle properties. The direct precipitation of lignin nanoparticles from wheat straw organosolv extracts can drastically reduce solvent consumption in a lignin nanoparticle production process. Processes shown so far in literature require extensive lignin purification and repeated precipitation of the lignin if the pretreatment process is included. The process shown in this work achieves the direct precipitation of lignin nanoparticles from the pretreatment extracts and; therefore, a drastic simplification of the process.

Particles with dimensions ranging from 97.3 to 219.3 nm could be produced and carbohydrate impurities reached values as low as in purified lignin particles at certain precipitation parameters. The results presented in this work can be used to optimize precipitation parameters with an emphasis on particle size, carbohydrate impurities or the solvent consumption in a straightforward process design.

## Supplementary Materials

The following are available online at https://www.mdpi.com/1420-3049/25/6/1388/s1, Table S1. Composition of the organosolv extract applied in the precipitation experiments, Table S2. Analysis of variance for fitted model for the carbohydrate and particle size response, Table S3. Coefficient values of the fitted model for the carbohydrate and particle size response.
