# Supplementary material for: Direct Precipitation of Lignin Nanoparticles from Wheat Straw Organosolv Liquors Using a Static Mixer
Source: Molecules. 2020 Mar 18;25(6):1388. doi: 10.3390/molecules25061388 (PMC7145315; doi:10.3390/molecules25061388)
Supplement: Supplementary file 1 [file molecules-25-01388-s001.pdf]

molecules

Article

Supplementary Materials:

Direct Precipitation of Lignin Nanoparticles from Wheat Straw Organosolv Liquors using a Static Mixer

Stefan Beisl *, Johannes Adamcyk and Anton Friedl

Institute of Chemical, Environmental and Bioscience Engineering, TU Wien, 1060 Vienna, Austria;
* Correspondence: stefan.beisl@tuwien.ac.at; Tel.: +43-1-58801-166258

Table S1. Composition of the organosolv extract applied in the precipitation experiments

Compound/Property	Value	Unit
Ethanol	511	g/l
Total Carbohydrates [1]	0.677	g/l
Monomeric Carbohydrates [1]	0.201	g/l
Acetic Acid	1.43	g/l
Acid Insoluble Lignin	5.53	g/l
Acid Soluble Lignin	1.09	g/l
Density [2]	0.901	g/ml
Dry Matter [3]	1.57	wt%

[1] Sum of arabinose, galactose, clucose, xylose and mannose concentrations; [2] at 25°C; [3] determined at 105°C

Table S2. Analysis of variance for fitted model for the carbohydrate and particle size response.

Response	Source	df	Sum of squares	Mean squares	F value	P value	R^2	Adj R^2
Carbohydrates	Regression	6	25.88	4.31327	37.25	<0.0001	0.89	0.87
	Residual	27	3.126	0.1158				
	Total	33	29.01					
Particle Size	Regression	9	48153	5350.34	28.98	<0.0001	0.92	0.88
	Residual	24	4431	184.6				
	Total	33	52585					

Table S3. Coefficient values of the fitted model for the carbohydrate and particle size response.

Factor	Carbohydrate Content				Particles Size			
	Coefficient	Standard Error	F value	P value	Coefficient	Standard Error	F value	P value
Constant	2.340	0.3718			304.4	15.94		
pH	0.1954	0.1020	3.670	0.06590	ns			
Ratio	ns				20.96	10.33	4.120	-0.054
Flowrate	ns				-2.836	0.5183	29.93	<0.0001
pH^2	ns				ns			
pH × Ratio	ns				-15.39	4.521	11.59	0.0023
pH × Flowrate	-0.0008020	0.0003888	4.250	0.04890	0.5192	0.1973	6.930	0.015
$Ratio^2$	ns				ns			
Ratio × Flowrate	-0.002065	0.0004693	19.36	0.00020	-0.05222	0.01510	11.96	0.0020
$Flowrate^2$	ns				0.007281	0.001018	51.17	<0.0001
pH^2 × Ratio	-0.01348	0.0031679	18.10	0.00020	1.113	0.4496	6.130	0.021
pH^2 × Flowrate	ns				-0.04888	0.01959	6.230	0.020
pH × $Ratio^2$	0.01031	0.0020067	26.41	<0.0001	0.4435	0.1091	16.51	0.00040
pH × Ratio × Flowrate	0.001	0.0001092	39.40	<0.0001	ns			

not significant (ns)
